# Transjugular Intrahepatic Porto-Systemic Shunt (TIPS), Mesenteric Vein Recanalization, and Rectal Varices Embolization Following Unsuccessful Endoscopic Management: A Case Report and Literature Review

**DOI:** 10.7759/cureus.83581

**Published:** 2025-05-06

**Authors:** Sayantan Patra, Sabharisundaravel Paulraj, Anadi Gupta, Rohit K Khandelwal, Shuvro H Roy Choudhury

**Affiliations:** 1 Interventional Radiology, Rabindranath Tagore International Institute of Cardiac Sciences, Kolkata, IND; 2 Interventional Radiology, Narayana Health, Kolkata, IND

**Keywords:** bleeding rectal varices, embolization/embolotherapy, endoscopic management, endovascular management, tipss, trans-tipss approach, uncompensated chronic liver disease

## Abstract

Rectal varices are rectal submucosal porto-systemic collaterals that develop secondary to portal hypertension in chronic liver disease. Bleeding from rectal varices is rare but potentially life-threatening. The management is typically endoscopic, with endovascular and surgical options for refractory cases. We present a case of endovascular salvage in a 58-year-old male patient with rectal varices causing life-threatening hematochezia, unresponsive to endoscopic procedures. Extensive rectal varices secondary to mesenteric venous obstruction were successfully managed with transjugular intrahepatic porto-systemic shunting (TIPS), mesenteric vein revascularization, and variceal glue embolization. Immediate technical and clinical success was followed by a long recurrence-free interval. The report highlights the importance of image-guided interventions for recalcitrant rectal variceal bleeding.

## Introduction

Rectal varices are porto-systemic collaterals in the rectal submucosa that develop due to portal hypertension, with the highest incidence being reported in extrahepatic portal vein obstruction (EHPVO) [1,2]. An accepted anatomic definition is when varices originate >4 cm above the anal verge [3]. Rectal varices are prevalent in cases of chronic liver disease (CLD) (40-77%) [4]. Bleeding rectal varices can be life-threatening, albeit rare (0.5-5%) [5]. However, a well-thought-out protocol for optimally managing this rare entity is yet to be developed, although a loosely woven hierarchy for specialized management does exist. The specialty of treatment and the modality of management mainly depend on the primary physician and the availability of specialized services rather than any other consideration [1].

## Case presentation

A middle-aged, 58-year-old man with chronic liver disease (non-alcoholic fatty liver disease, NAFLD-related) was referred for recalcitrant hematochezia, resistant to multiple sessions of endoscopic treatment. Prior to the referral, he had been evaluated with contrast CT on previous occasions.

Upon presentation, the patient had hemoglobin levels of 5.4 g/dL (even after multiple blood transfusions), was classified as Child-Pugh B, and had a MELD (model for end-stage liver disease) score of 14 points. The consideration for an urgent liver transplant was negated due to a poor surgical profile. Hence, based on multi-disciplinary consensus, the patient was scheduled to undergo endovascular management of portal vasculature after a therapeutic single-time aspiration of ascites to relieve respiratory and abdominal discomfort.

Imaging findings

**Figure 1:**
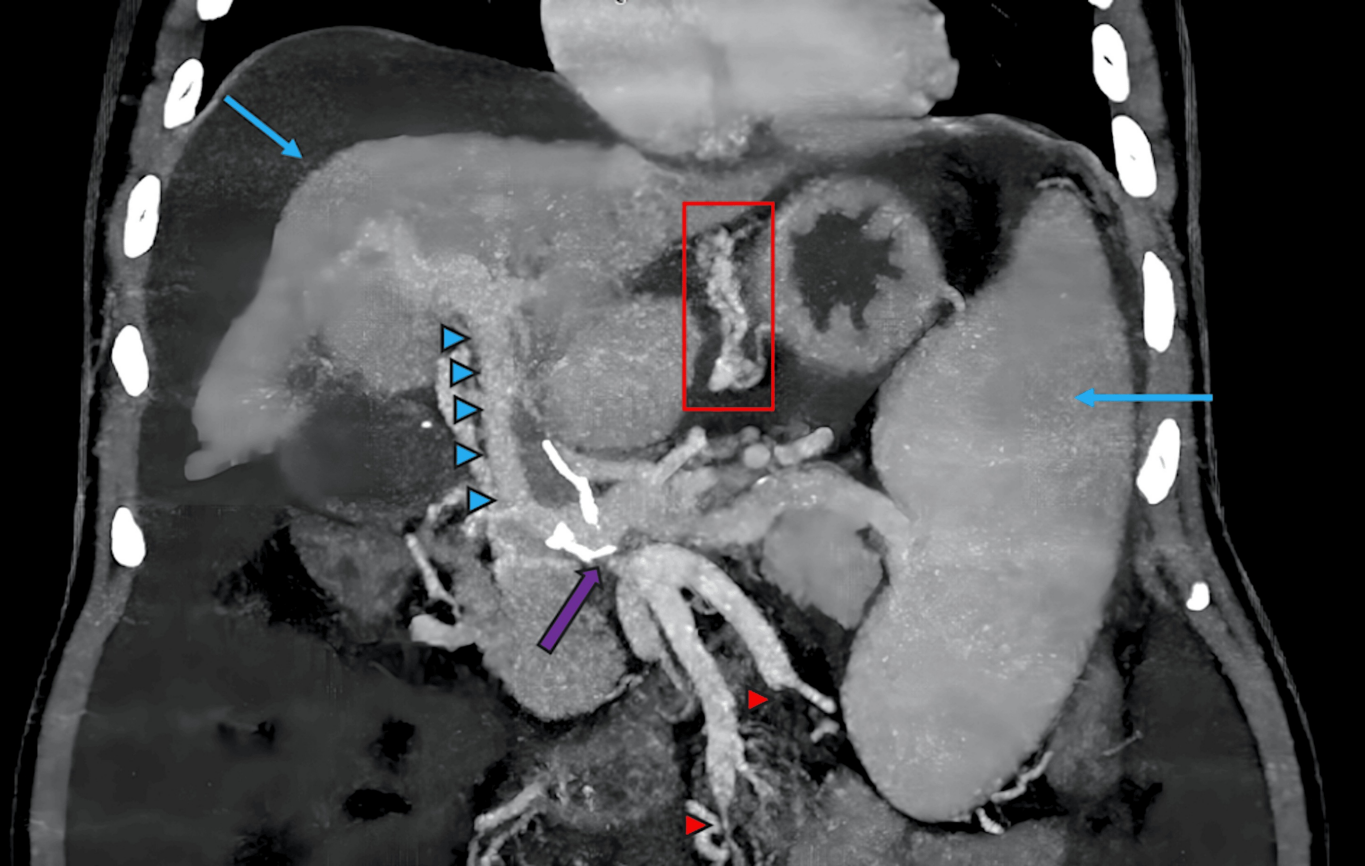
Pre-procedure contrast-enhanced computed tomography image The image shows the classical signs of chronic liver disease—nodular liver with right lobe atrophy and splenomegaly (blue arrows). Extra-mural gastric varices are marked with a red rectangle. Portal hypertension is marked with blue arrowheads; signs of portal hypertension are readily apparent. The SMV-IMV conjoint trunk ostio-proximal stenosis is indicated by a purple arrow. Both SMV and IMV show chronic thrombus and short-segment CTO (red arrowheads). These were later confirmed with diagnostic digital subtraction angiography. SMV: superior mesenteric vein; IMV: inferior mesenteric vein; CTO: chronic total occlusion

**Figure 2:**
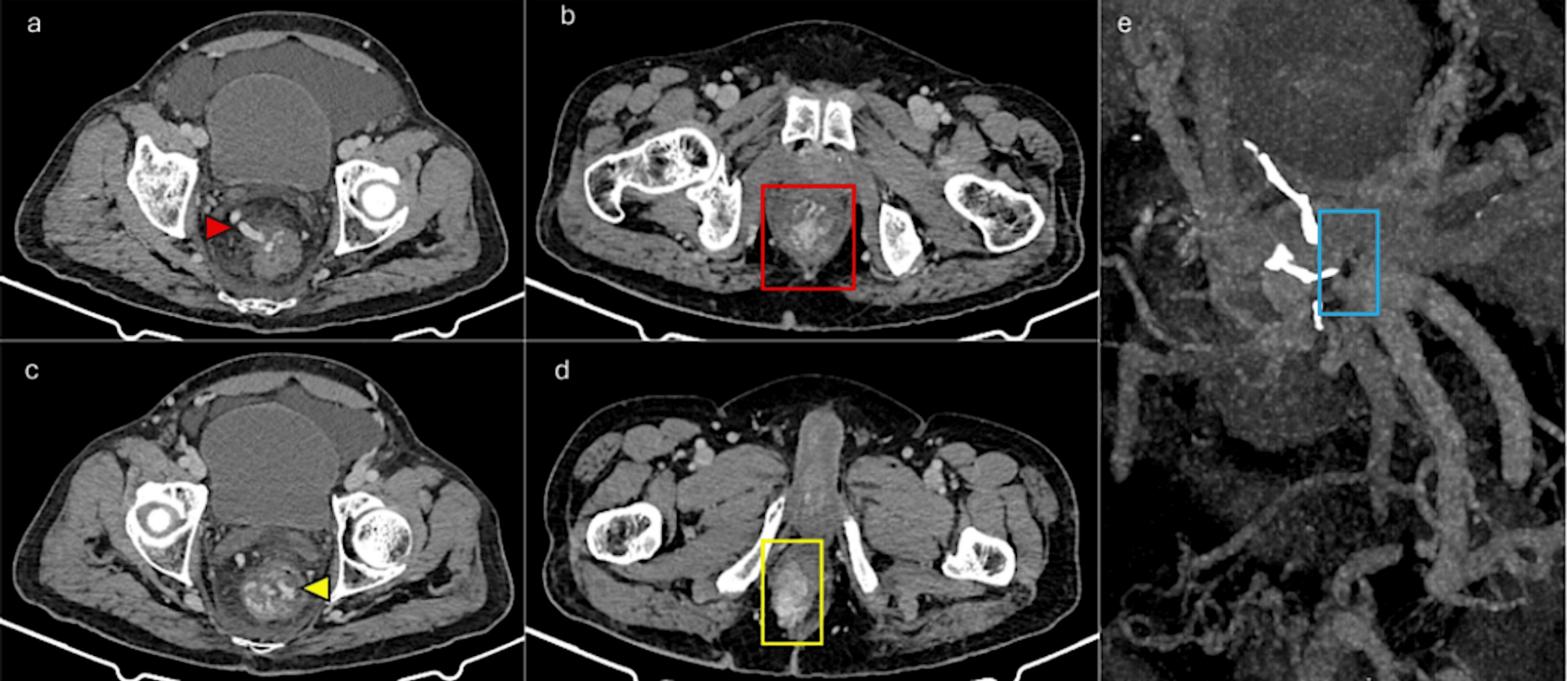
Pre-procedure computed tomography image The image shows (a) a dilated and tortuous IMV (red arrowhead) supplying the rectal varices in (b) (red box); (c) active extravasation (yellow arrowhead) and prolapse of the ano-rectal complex are also apparent (d) (yellow box). (e) CT images show stigmata of portal hypertension with severe ostio-proximal stenosis of the SMV and IMV (blue box). These were later confirmed with diagnostic digital subtraction angiography. IMV: inferior mesenteric vein; SMV: superior mesenteric vein; CT: computed tomography

A CT review confirmed extensive rectal varices as the source of the bleed. The underlying cause of the rectal varices was postulated based on CT (and later confirmed on angiograms) to be stenosis of the superior mesenteric vein (SMV) and short-segment chronic occlusion of the proximal inferior mesenteric vein (IMV). There were multiple resultant porto-portal and porto-systemic collaterals. The rectal varices were found to arise from the terminal recto-sigmoid branches of the IMV. The working hypothesis was that rectal varices were arising due to a lack of drainage into the mesenteric veins and resultant back pressure (Figures 1, 2) (Video 1).

**Video 1:** Axial scroll video of pre-procedure computed tomography scan An axial CT scroll video confirms the findings of the CT images. Video Credits: Anadi Gupta

Management

**Figure 3:**
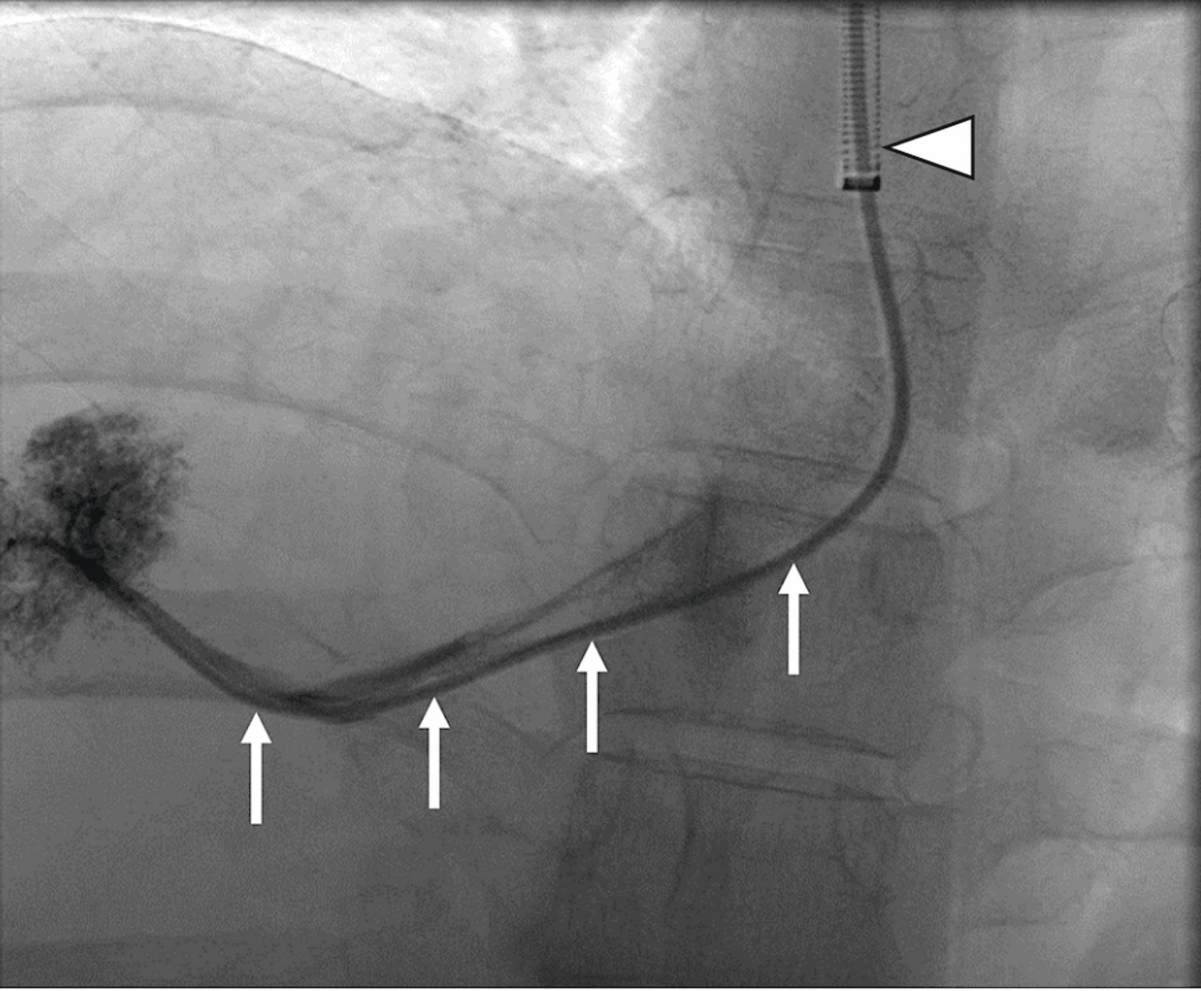
Hepatic vein catheterization Catheterization of the right hepatic vein with a multi-purpose angled (MPA) catheter (white arrows) and a long sheath (arrowhead).

After a standard pre-procedure workup and pre-anesthetic evaluation, the patient underwent an emergent endovascular procedure. Right internal jugular access was secured with a standard 12 Fr 11 cm short sheath, which was exchanged for a Cook® long sheath (Cook Medical, Chennai, India). This was followed by the right hepatic vein catheterization with a multipurpose angiographic catheter and 0.035'' Terumo® Glidewire™ (Gurugram, India) (Figure 3).

Glidewire™ was exchanged for an Amplatz™ stiff wire (Boston Scientific, Gurugram, India), and a Cook® Ring Transjugular Intrahepatic Access Set™ was used to puncture from the right hepatic vein access to the right portal vein under fluoroscopic and ultrasound guidance (Video 2).

**Video 2:** Portal vein access Puncture from the right hepatic vein (RHV) to the right portal vein (RPV) using a tri-axial Transjugular Intrahepatic Access^TM^ set. Video Credits: Anadi Gupta

**Figure 4:**
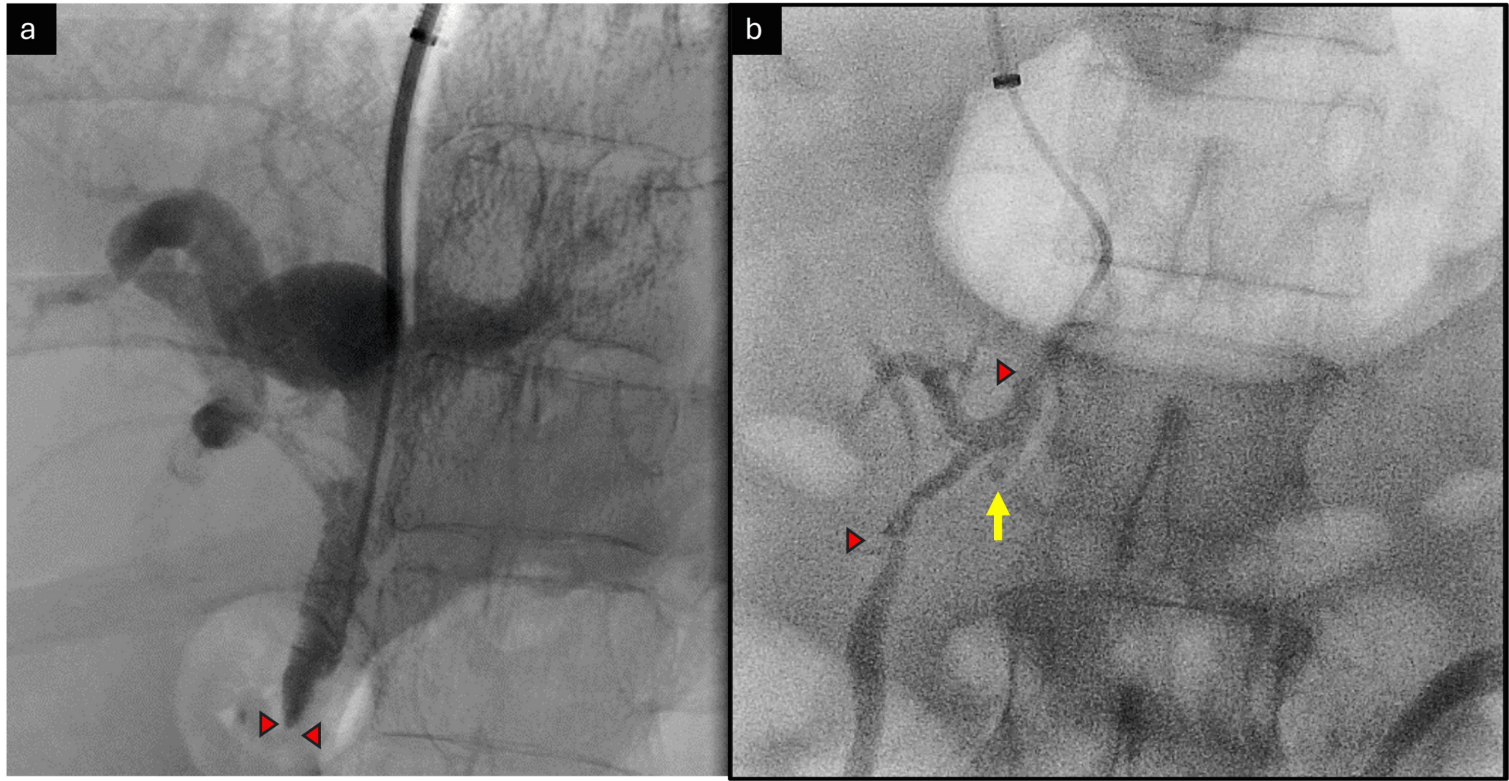
Crossing the SMV stenosis Steno-occlusive disease of SMV at the ostio-proximal segment is shown with red arrowheads (a) before and (b) after crossing the stenosis. Note the IMV stump (yellow arrow). SMV: superior mesenteric vein; IMV: inferior mesenteric vein

An angiography was subsequently performed to confirm mesenteric venous obstruction. The SMV stenosis was crossed with a right-angled catheter and Artivion® E-wire™ (New Delhi, India). The wire was positioned in mid-SMV. This was followed by control runs and angiograms to confirm the position of the stenoses in the ostio-proximal segment (Figure 4).

**Figure 5:**
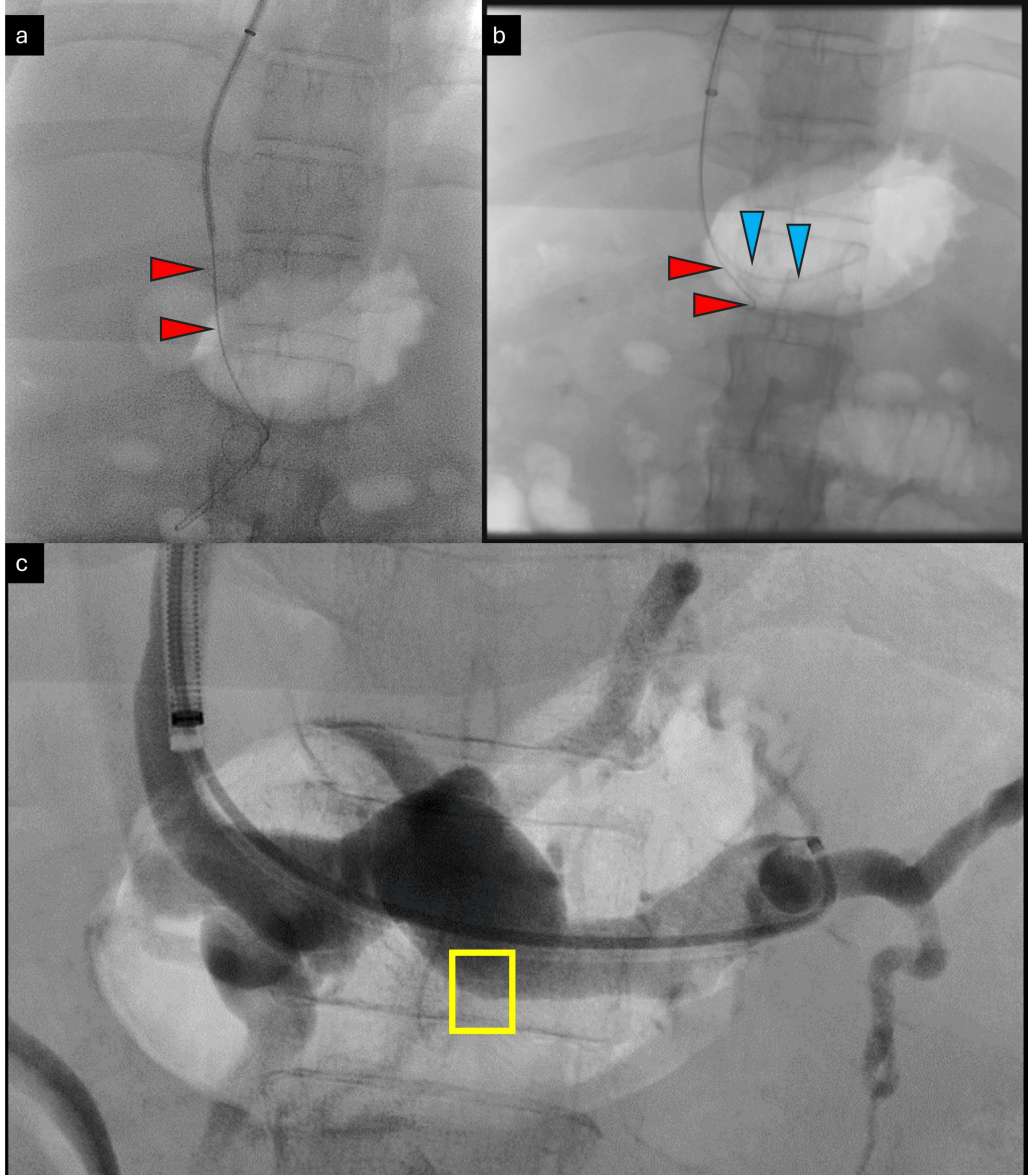
Splenoportal axis access and angiogram Fluoroscopic and digital subtraction angiogram showing (a) a safety wire placed in the SMV (red arrowheads), (b) wire and catheter in the splenic vein (blue arrowheads), and (c) small conical stump (nubbing) of IMV in the splenic vein (yellow box). SMV: superior mesenteric vein; IMV: inferior mesenteric vein

With a safety wire in the SMV, selective catheterization of the IMV was attempted after angiograms and control runs from the splenic vein. Diagnostic intra-procedural imaging showed a small conical stump at the ostium of the IMV (Figure 5).

After exhaustive attempts using different catheters and wire combinations, the IMV could be finally catheterized with sharp recanalization using the back end of a soft 0.035" Terumo® Glidewire™ (Video 3).

**Video 3:** Sharp recanalization of the IMV Multiple attempts to cross the inferior mesenteric vein (IMV) were unsuccessful, while sharp recanalization was ultimately successful. Video Credits: Anadi Gupta

**Figure 6:**
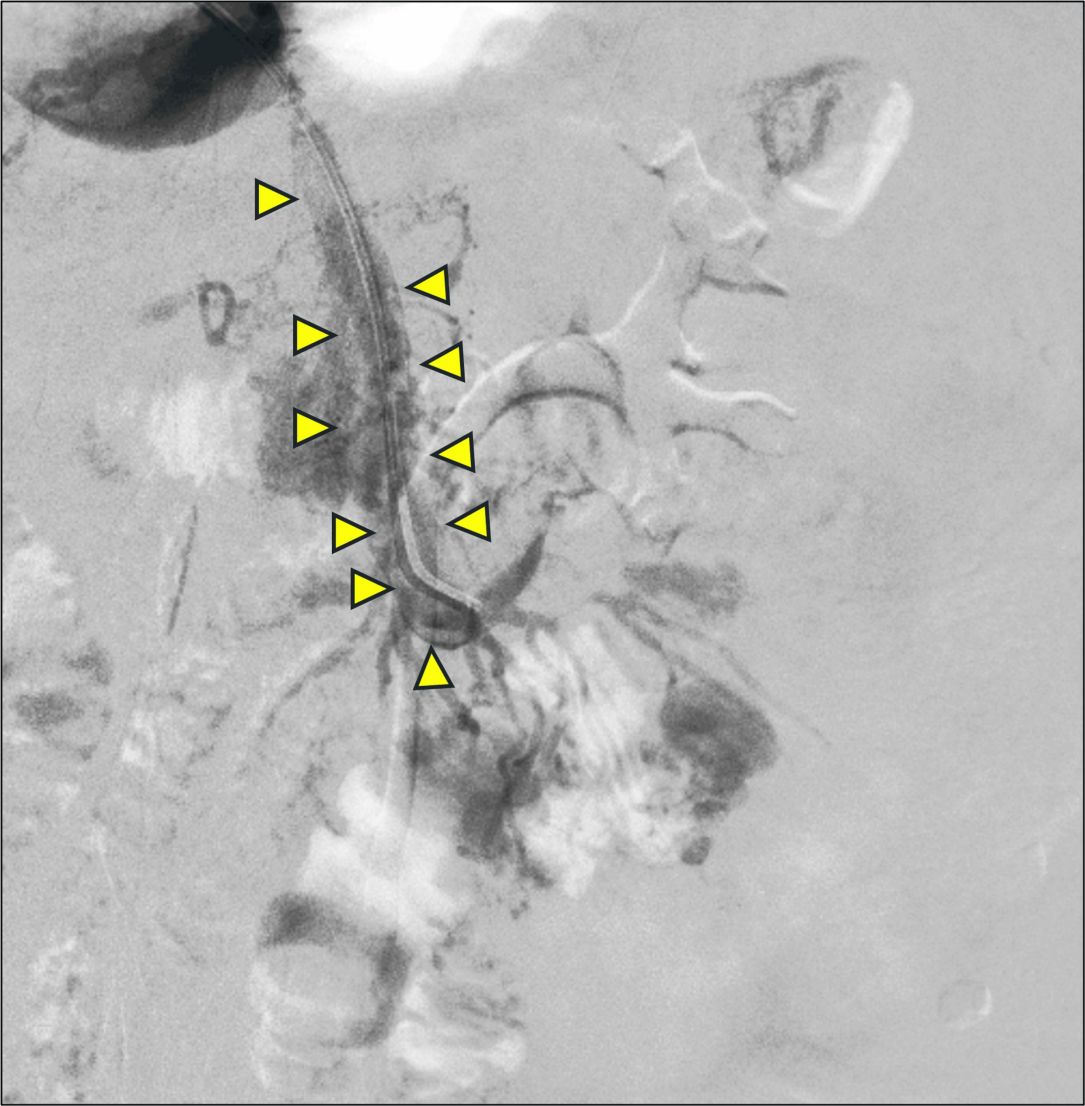
Inferior mesenteric vein angiogram The inferior mesenteric vein angiogram shows thrombus (filling defects, marked with yellow arrowheads), which was later laced with recombinant tissue plasminogen activator (Alteplase).

Following catheterization, control runs and angiograms showed acute on chronic thrombosis of the IMV. The IMV was laced with 6 mg recombinant tissue plasminogen activator (rTPA) (Figure 6).

**Figure 7:**
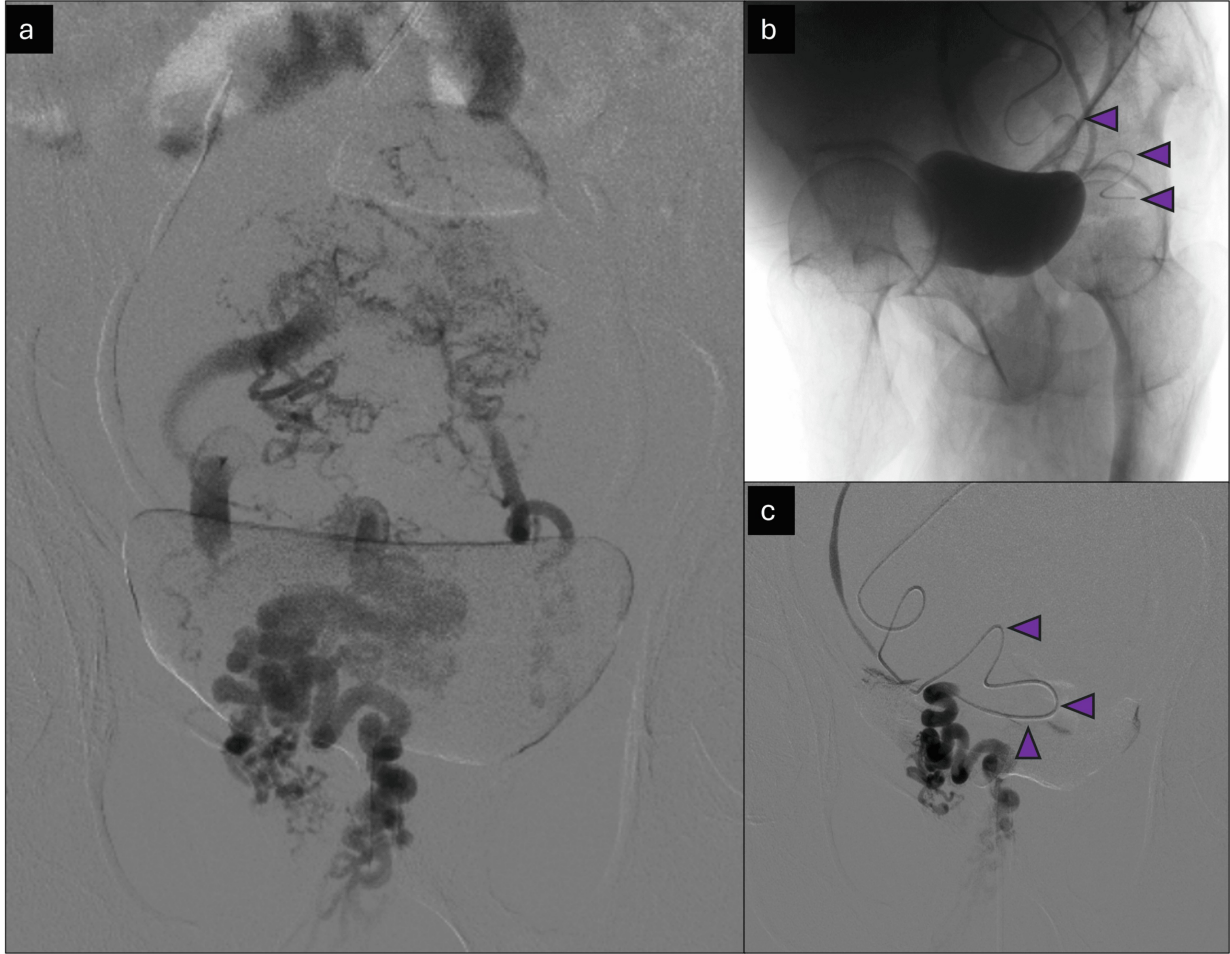
Variceal angiogram The image shows a digital subtraction angiogram of the varices. (a) Selective angiograms from the rectosigmoid branch of the IMV showing extensive rectal varices (convoluted network of veins); (b) super-selective microcatheter positioning (marked with purple arrowheads); (c) super-selective angiography with a microcatheter prior to glue embolization.

This allowed further intervention through the IMV. The wire and catheter combination was passed distally into the terminal IMV up to the level of the rectosigmoid branches. Control runs and angiograms at this level showed the rectal varices in their entirety. Bilateral rectal branches were super-selectively catheterized with a microcatheter, and super-selective angiograms were obtained (Figure 7).

**Figure 8:**
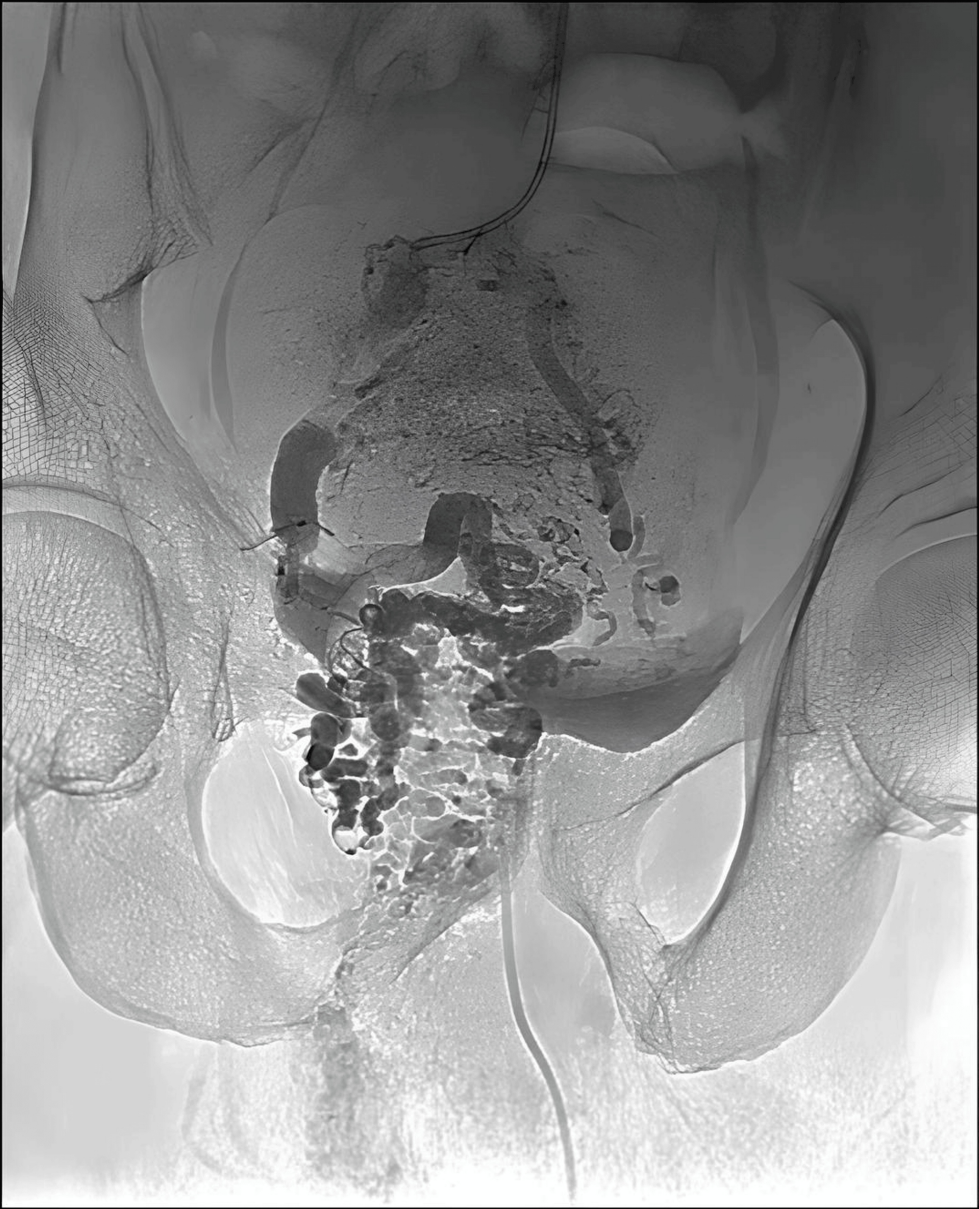
Final glue cast A single-shot image (no contrast injected) shows the final glue cast (opacified arteries) in the pelvic varices.

With the final position of the microcatheter in the trunks of the rectal branches of the IMV, a 1:4 cyanoacrylate glue:Lipiodol™ mixture was injected into the rectal varices until contrast backflow was noted (Video 4). The completion angiogram after embolization showed excellent results with backflow of contrast in the trunk of the rectal vessels and a continuous column of glue cast within the embolized vessels (Figure 8).

**Video 4:** Injection of a 1:4 cyanoacrylate glue-Lipiodol™ mixture into the rectal varices until contrast backflow was noted Video Credits: Anadi Gupta

**Figure 9:**
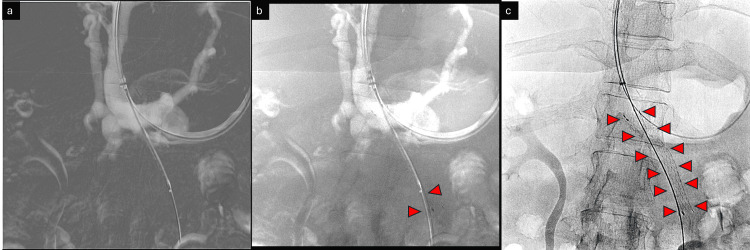
Superior mesenteric vein stenting Images showing stenting of the superior mesenteric vein (SMV). (a) Positioning of an undeployed stent under Road-Map guidance; (b) deployment of stent under Road-Map guidance (distal deployed struts marked with red arrowheads); (c) fully deployed stent (single-shot radiograph image, deployed stent marked with red arrowheads).

In the next step, the SMV was stented with a 10 mm × 60 mm uncovered stent (Bard® E-Luminexx^TM^, Bard Peripheral Vascular, Inc., Mumbai, India) over a stiff safety wire already in position. Subsequently, this was balloon molded with a 10 mm × 40 mm non-compliant balloon (Figure 9).

**Figure 10:**
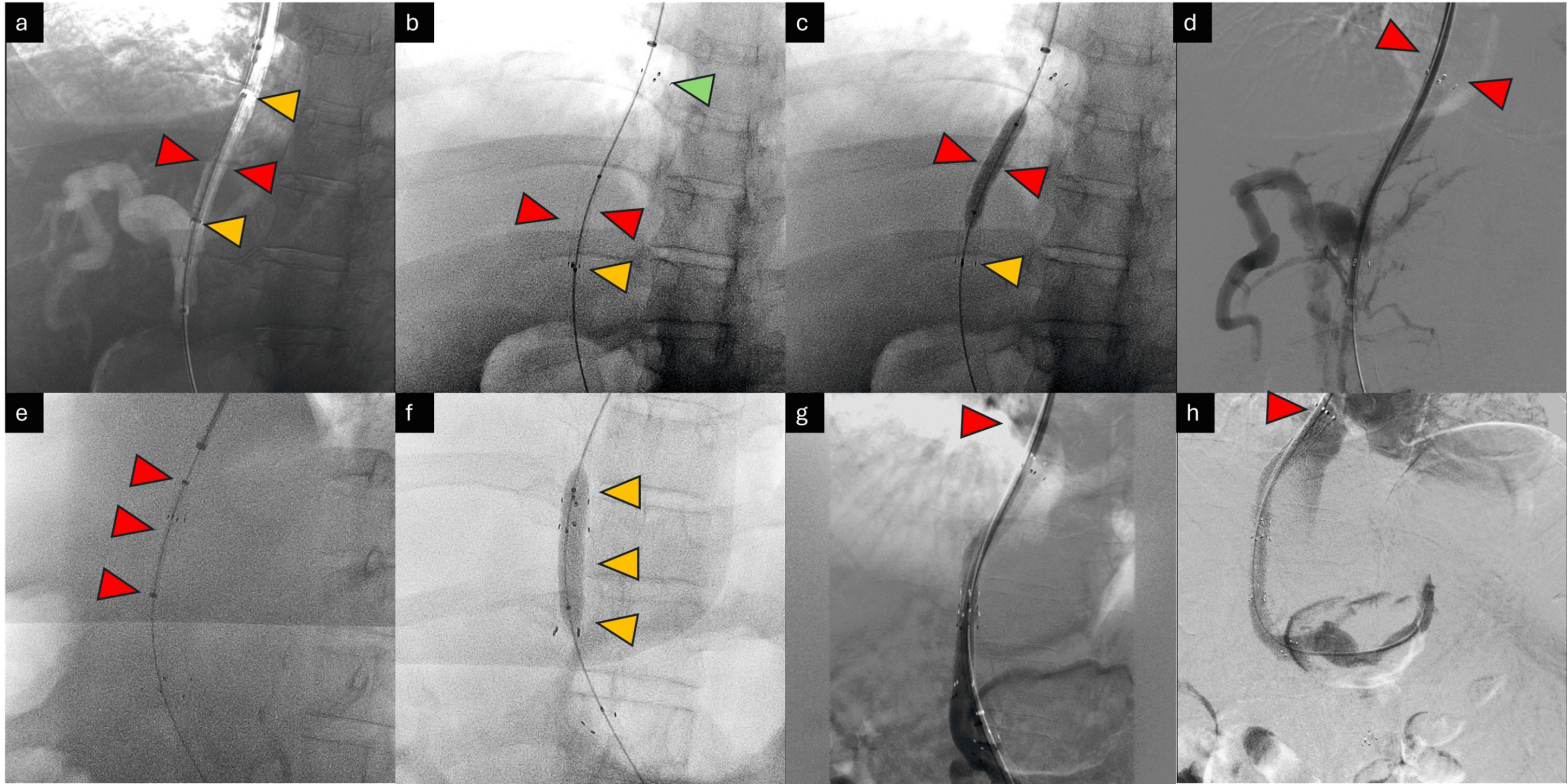
Transjugular intrahepatic porto-systemic shunting and completion angiogram Images depicting TIPS and completion angiogram. (a) Positioning of covered stent (10 × 80 mm), depicted with red arrowheads, under Road-Map guidance. Note that the edge markers are marked with yellow arrowheads. (b) The deployed covered stent was only partially open in the inferior aspect. The covered stent is marked with red arrowheads. The proximal open end is marked with a green arrowhead. The distal stent markers, which remained unopened, are marked with yellow arrowheads. (c) Balloon molding of the stent graft is done. Struts in the distal part are seen to open. The inflated balloon is marked with red arrowheads. The distal open end is marked with yellow arrowheads. (d) The angiogram shows sub-optimal results, with poor opacification of the IVC, and is marked with red arrowheads. (e, f) The deployment and balloon molding of a second stent graft (10 × 40 mm) as an extension of the previous stent graft. The stent graft and balloon are marked with red and yellow arrowheads, respectively. (g, h) The completion angiograms showcase excellent results. Opacification of the IVC is shown with red arrowheads. TIPS: transjugular intrahepatic porto-systemic shunting; IVC: inferior vena cava

As the final step, a 10 mm × 80 mm stent graft was initially deployed from the middle hepatic vein to the RPV. However, due to some foreshortening, this had to be extended with a 10 mm × 40 mm stent graft (Bard® Fluency™). The transjugular intrahepatic porto-systemic shunting (TIPS) stents were subsequently balloon molded with appropriately sized balloons. The final completion angiogram with the tip of the catheter in the SMV showed excellent results with the presence of expected flow from the SMV to the portal vein into the middle hepatic vein and finally into the inferior vena cava (IVC) and right atrium (Figure 10).

Follow-up

Technical success was followed by clinical success (complete cessation of hematochezia), albeit with the development of grade I hepatic encephalopathy, which was conservatively managed. The patient presented for follow-up after six months. On follow-up CT, there was adequate flow through the TIPS stent graft. Additionally, there was good flow through the IMV and SMV. Porto-portal and porto-systemic collaterals had reduced considerably. An excellent glue cast was seen in the embolized rectal varices. There was no recurrence of bleeding per rectum, although the patient frequently had symptoms of mild encephalopathy.

**Figure 11:**
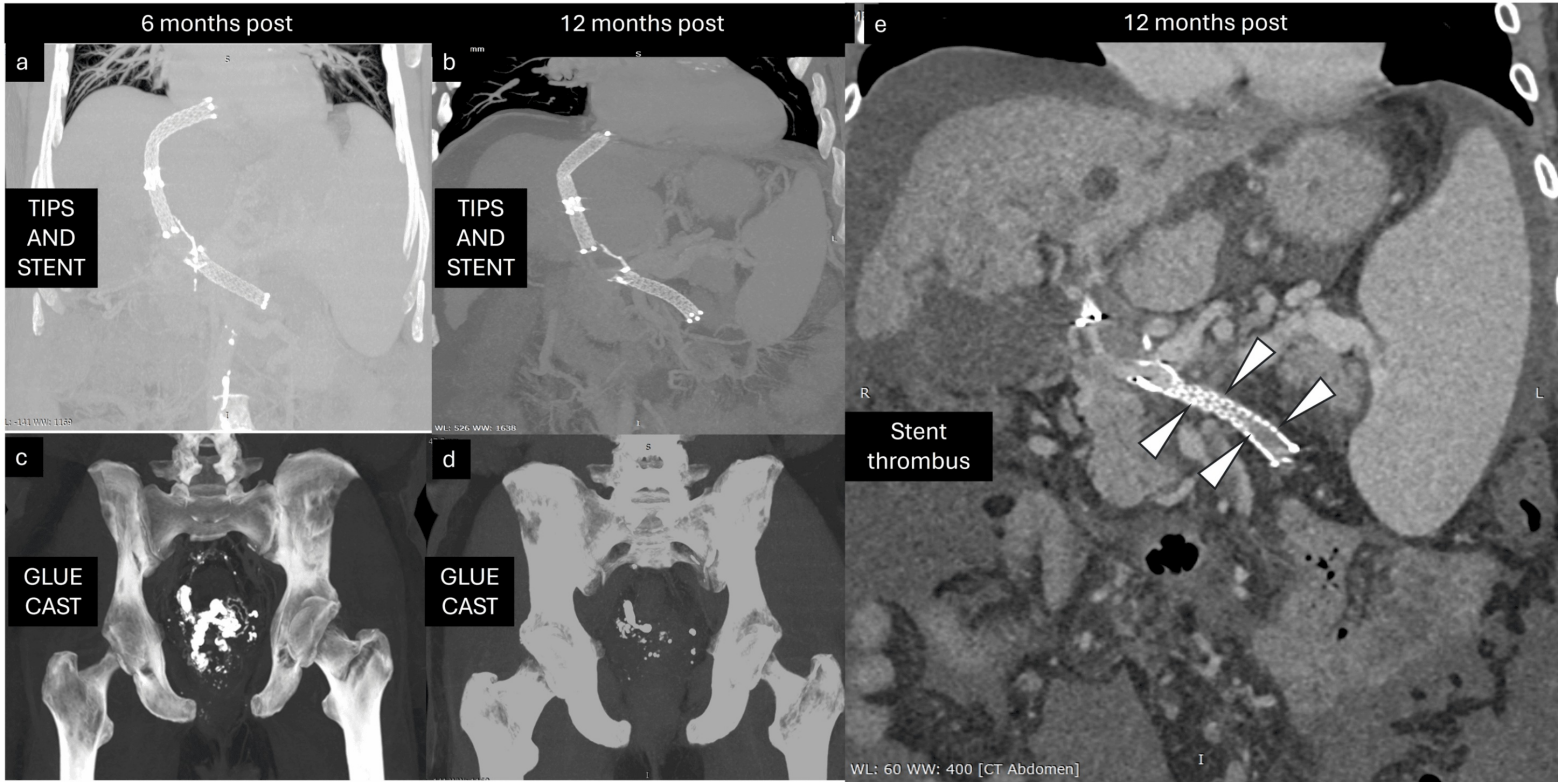
Follow-up contrast computed tomography images Images from bi-annual follow-up computed tomography: (a) and (c) are six months post-CT images showing expected positions of the stent and glue cast, respectively. (b) and (d) are 12 months post-CT images showing a slightly deformed superior mesenteric vein (SMV) stent. (e) Deformed SMV stent with distal thrombus (white arrowheads).

On further follow-up, one year after the procedure, the patient presented with mild hepatic encephalopathy. A repeat CT showed a patent TIPS shunt (Figure 11). However, there was partial thrombosis of the SMV stent with distal flow through collaterals. The IMV was chronically thrombosed. Although unfortunate, the absence of symptoms ensured no further active intervention was required. The patient improved on conservative management and was discharged with advice for restricted domiciliary activity. On telephonic follow-up, the patient has considerably improved physically and resumed regular activities.

## Discussion

Demographics and etiology

Rectal varices are porto-systemic collaterals in the rectal submucosa that develop due to portal hypertension, with the highest incidence being reported in EHPVO [1,2]. An accepted anatomic definition is when varices originate >4 cm above the anal verge [3]. Rectal varices are common in settings of CLD (40-77%) [4]. Bleeding rectal varices can be life-threatening, albeit rare (0.5-5%) [5]. However, a well-thought-out protocol for optimally managing this rare entity is yet to be developed, although a loosely woven hierarchy for specialized management does exist. The specialty of treatment and the modality of management mainly depend on the primary physician and the availability of specialized services rather than on any other consideration [1].

Treatment and prognosis of bleeding rectal varices

The accepted first-line management after emergent stabilization, blood transfusion, and correction of coagulopathy is unequivocally endoscopic, with sclerotherapy being considered superior to banding/ligation [6,7]. The literature widely reports that this is successful in most cases, with the advantage of being minimally invasive and requiring the least pre-procedure preparation [2].

Endovascular procedures are considered second-line, indicated for unsuccessful endoscopic interventions or recalcitrant bleeds. Among endovascular procedures, TIPS with or without antegrade embolization is the mainstay of treatment with minor procedural or technical variations among different individuals and institutes [8]. Recent reports of successful retrograde obliteration (balloon-assisted retrograde transvenous obliteration (BRTO)/coil-assisted retrograde transvenous obliteration (CARTO)/plug-assisted retrograde transvenous obliteration (PARTO)) have also emerged, which may potentially prove to be a valuable option in the future [9]. Surgical procedures are considered the last resort when all other options have been exhausted, although patients with clinically significant hematochezia are not the best surgical candidates [10].

The first report on bleeding rectal varices dates back to 1954 by Cabot et al. [3,11]. Rectal varices in chronic liver disease are not uncommon. The development of rectal varices correlates positively with an increase in HVPG (hepatic venous pressure gradient) beyond 12 mmHg as well as higher CTP (Child-Turcotte-Pugh) and MELD scores [12]. Controversy exists regarding the correlation between the development of rectal varices and the successful management/closure of gastroesophageal varices. Some authors theorize that obliteration of the upper gastrointestinal collateral pathways favors the development of collaterals from the IMV or superior rectal vein and therefore rectal varices [1].

Clinical and imaging findings of bleeding rectal varices

Endoscopy is the primary diagnostic modality for rectal varices. Varices typically appear white or blue, tortuous or coil-like, with or without overlying red telangiectatic lesions. In addition, active bleeds and mucosal erosion/ulcer/scar may also be seen [13]. Endoluminal evaluation can be augmented with endoscopic ultrasound (EUS) for peri-rectal collaterals. Color Doppler using EUS can be used to indirectly categorize the risk of bleeding through numerical parameters [1].

The role of radiological imaging cannot be understated. The imaging appearance of rectal varices is similar to that of hemorrhoids. However, varices typically occur above the level of the dentate line. The most common findings are serpiginous dilated veins surrounding (para-rectal) and within (rectal) the rectum. Traditionally, any gastrointestinal bleed has been diagnosed with either radionuclide scintigraphy or catheter angiography. To overcome the significant limitations of either of these two, computed tomography and, in limited scenarios, magnetic resonance imaging are the current reference standards. Provision of luminal and extra-luminal information and detection of bleeding as low as 0.35 mL/minute (with multidetector computed tomography angiography, MDCT) are the principal advantages of cross-sectional imaging modalities [14,15]. In acute settings, multiphasic CT acquisition is the investigation of choice for bleeding rectal varices. Additionally, maximum intensity projection and volume rendering not only help identify the site of the bleeding but also help in intervention planning [16]. In this case, active extravasation could be appreciated because of the heavy bleeding, although it is not a common occurrence.

Multimodality management

Portal hypertension causing rectal variceal bleed is common and effectively managed with conservative and/or colonoscopic means. Conservative management of bleeding rectal varices involves volume repletion with fluids and blood transfusions. Notwithstanding the lack of randomized control trials, there is no proven role of pressor agents [1,17]. Endoscopic sclerotherapy or band ligation is the primary management. Among these, sclerotherapy has a lower recurrence rate and incidence of complications [18]. Glue injection is not FDA approved, but it is effective when used in conjunction with endoscopic ultrasound. Serious adverse events include systemic embolization and sepsis, such as stroke and organ infarction through a patent foramen ovale. Endovascular management is the second-line intervention and includes either TIPS with embolization or BRTO, which are discussed in the following text.

Traditionally, surgeries were reserved for failed conventional management. Surgical methods include simple suture, staple ligation, or IMV occlusion [19,20]. In extremis, management involves portacaval shunt surgery. Regardless, most patients who have clinically significant rectal variceal bleeding are not good candidates for major surgical procedures, with mortality as high as 80% [1,19].

Role of interventional radiology in intractable variceal bleed

The incidence of intractable bleeding in rectal varices is rare [21,22]. Interventional radiology management is indicated only when the bleed is refractory to or in the presence of a contraindication to other more conventional procedures/approaches, such as endoscopy [1]. The first-line intervention for recalcitrant bleeding from rectal varices is TIPS. The efficacy of TIPS in controlling rectal variceal bleed is, however, as yet unproven by large-scale RCTs [23]. TIPS can even be placed under intravascular ultrasound (IVUS) guidance in difficult targeting or small portal veins. Commonly, this is enough to resolve the high portal venous pressure causing the rectal varices.

Directed interventions for rectal varices are required in specific cases, as TIPS may not be able to control bleeding in all cases [24]. Techniques such as BRTO or BATO (balloon-assisted antegrade transvenous obliteration) are commonly used, which are basic variants of balloon-assisted embolotherapy with liquid embolics (usually a 5-F Fogarty balloon or even non-compliant balloons). Sclerosant or other liquid embolic (cyanoacrylate glue or ethylene vinyl alcohol) is injected into the varices with a half-deployed coil in either the portal or systemic vein, followed by the completion of coil deployment after the injection is over. Vascular plug or coil variants of this technique are also well described [25]. Although TIPS is the preferred route, transhepatic or, on occasion, transsplenic variceal embolization is also a viable option, particularly in patients with poor performance status and a history of prior (high-grade) hepatic encephalopathy [24]. In our case, we used an unusual variation of these techniques (also described in prior literature) along with IMV stenting to prevent recurrence [24]. In our case, a balloon was not required as the forward flow was toward the systemic veins due to the occlusion of the portal vein.

In a study by Mansour et al. in 2022, 21 patients with portal vein thrombosis were included for interventional radiology management [21]. Portal vein thrombosis in nine patients was treated by percutaneous technique (PCT) and in 12 by TIPS. The difference in technical success (55.5% with PCT vs. 83.3% with TIPS) was not statistically significant. The creation of TIPS provided statistically significant better outcomes (86.6% vs. 33.3%). Multivariate analysis revealed that thrombolysis and percutaneous access posed a high risk of bleeding. In our study, we successfully employed thrombolysis without any adverse consequences. Since TIPS provided optimal results in this previous study, we opted for this route and not the less invasive transhepatic route [26]. A comparison of TIPS vis-à-vis transhepatic access is summarized in Table 1.

**Table 1:** Comparison of access routes for portal vein thrombosis recanalization Summary of the key differences between percutaneous and trans-TIPS PVT (portal vein thrombosis) recanalization.

Parameter	Percutaneous Route	Trans-TIPS
Principle	Preservation of the endothelial wall and physiological blood flow through the native portal vein and intrahepatic branches	Creation of an artificial trans-parenchymal connection. The tract has subnormal endothelialization
Difficulty in intrahepatic PV thrombosis	Difficult due to limited flow	Easier and also allows access to the porto-mesenteric system
Access for further intervention if required	Difficult	Easy

## Conclusions

Our case was unique due to a major and life-threatening rectal variceal bleed (which is a rare occurrence), refractory to multiple sessions of endoscopic therapy because it was precipitated by mesenteric venous obstruction (which again is unusual). It was managed by embolotherapy with glue rather than the conventionally used sclerosant, required sharp recanalization (which is sparsely described for mesenteric veins in literature, in contradistinction to central vein occlusions), and required mesenteric venous stenting after thrombolysis of the IMV. The trans-TIPS approach is common and regularly used. We could have used the trans-hepatic or trans-splenic approach in hindsight, keeping patient parameters in view. Fortunately, porto-portal collaterals ensured that the patient had no mesenteric ischemia related to the SMV stenosis and IMV thrombosis.

The incidence of mesenteric vein stent deformation and resultant in-stent restenosis and thrombosis is not uncommon and is commonly attributed to the hypermobility of the stented segment. Despite the unfortunate outcome, there were no adverse consequences until the last follow-up, likely due to well-developed collaterals resulting from the chronic nature of the event. Clinical and technical success was ensured by the adept execution of planned and unplanned issues during the intervention, the availability of a state-of-the-art cath suite and equipment, and excellent support staff.
